# Molecular Approaches for Detection of the Multi-Drug Resistant Tuberculosis (MDR-TB) in Bangladesh

**DOI:** 10.1371/journal.pone.0099810

**Published:** 2014-06-16

**Authors:** Tafsina Haque Aurin, Saurab Kishore Munshi, S. M. Mostofa Kamal, Md. Mostafizur Rahman, Md. Shamim Hossain, Thaythayhla Marma, Farjana Rahman, Rashed Noor

**Affiliations:** 1 Department of Microbiology, Stamford University Bangladesh, Dhaka, Bangladesh; 2 National Tuberculosis Reference Laboratory (NTRL), NIDCH, Mohakhali, Dhaka, Bangladesh; 3 University Research Co., LLC (URC), Banani, Dhaka, Bangladesh; Indian Institute of Science, India

## Abstract

The principal obstacles in the treatment of tuberculosis (TB) are delayed and inaccurate diagnosis which often leads to the onset of the drug resistant TB cases. To avail the appropriate treatment of the patients and to hinder the transmission of drug-resistant TB, accurate and rapid detection of resistant isolates is critical. Present study was designed to demonstrate the efficacy of molecular techniques inclusive of line probe assay (LPA) and GeneXpert MTB/RIF methods for the detection of multi-drug resistant (MDR) TB. Sputum samples from 300 different categories of treated and new TB cases were tested for the detection of possible mutation in the resistance specific genes (*rpoB*, *inhA* and *katG*) through Genotype MTBDRplus assay or LPA and GeneXpert MTB/RIF tests. Culture based conventional drug susceptibility test (DST) was also carried out to measure the efficacy of the molecular methods employed. Among 300 samples, 191 (63.7%) and 193 (64.3%) cases were found to be resistant against rifampicin in LPA and GeneXpert methods, respectively; while 189 (63%) cases of rifampicin resistance were detected by conventional DST methods. On the other hand, 196 (65.3%) and 191 (63.7%) isolates showed isoniazid resistance as detected by LPA and conventional drug susceptibility test (DST), respectively. Among the drug resistant isolates (collectively 198 in LPA and 193 in conventional DST), 189 (95.6%) and 187 (96.9%) were considered to be MDR as examined by LPA and conventional DST, respectively. Category-II and -IV patients encountered higher frequency of drug resistance compared to those from category-I and new cases. Considering the higher sensitivity, specificity and accuracy along with the required time to results significantly shorter, our study supports the adoption of LPA and GeneXpert assay as efficient tools in detecting drug resistant TB in Bangladesh.

## Introduction

Tuberculosis (TB), especially the drug resistant ones, appears as a major health problem worldwide with mortality ranging from 1.6 to 2.2 million per year [1], [2], [3]. TB also stands as a deadly issue in Bangladesh with the commencement of more than 350,000 new cases with an approximate estimation of 70000 deaths a year [3], [4], [5], [6]. The country ranks 6^th^ among the 22 TB burdened countries in the world and 9^th^ among 25 high priority multi-drug resistant (MDR) and extensively-drug resistant (XDR) TB flourished countries [3], [4], [7]. Several studies have been conducted till date to assemble the information on MDR- and XDR-TB situation, and to implement efficient methods for TB diagnosis in Bangladesh [3], [4], [5], [6], [7], [9], [10], [11]. However, the overall control of TB situation in this country is still inferior due to the lack of early and proper management of drug resistant TB cases.

Smear microscopy has long been known as the primary method for screening of TB, with a case detection rate of not more than 68% [12], [13]. A marked instance of TB misdiagnosis was evident in 2010 where 2 million of the 5.8 million (34.4%) globally notified cases were found to be smear negative [14]. Culture-based methods remain the “gold standard” for TB diagnosis in developing countries as these techniques have been greatly improved and routinely used over the past decade [15]. However, the time for a bacteriological culture-based diagnosis of TB may require several weeks to months [16], [17]. To address such delay in TB diagnosis as well as to discretely improve the diagnosis accuracy, molecular diagnosis aspects need to be considered for the early detection of *M. tuberculosis* which involves the detection of the mutation in specific genes imparting resistance against rifampicin (RIF) and/or isoniazid (INH), mostly used as the first line anti-tubercular drugs [18], [19].

Considerable advancement has been made in the last few years to resolve the basis of resistance against INH and RIF [19]. Although alterations in at least four genes has been found to be responsible for INH resistance, extensive studies have revealed that the resistance is likely to be associated with specific mutations in codon 315 of *katG* encoding catalase peroxidase and in the promoter region of *inhA* [19], [20], [21]. On the other hand, missense mutations, small deletions or insertions in the *rpoB* gene encoding the β-subunit of RNA polymerase are known to be responsible for the resistance against RIF [19], [20]. Several studies have pointed that more than 95% of RIF resistant strains shield a mutation within 81-bp core region of the *rpoB* gene [19], [20], [22], [23]. Detection of such mutations can be simply accomplished by implementing the line probe assay (LPA, to detect mutation in *rpoB*, *katG* and *inhA*) and GeneXpert (to detect mutation in *rpoB*) methods [24].

In 2008, World Health Organization (WHO) endorsed GenoType MTBDR*plus* (version 1.0) line probe assay (LPA), which is a rapid detection procedure of *Mycobacterium tuberculosis* complex (MTB) and also serves to detect mutations in the resistance specific genes conferring resistance against RIF and INH in AFB smear-positive sputum specimens [24], [25], [26]. The GeneXpert MTB/RIF assay was also implemented by WHO in 2010 to facilitate the rapid detection of *M. tuberculosis* isolates as well as to detect the resistance against RIF with a revelation of more than 95% sensitivity for smear-positive cases and 55% for smear-negative cases [24], [27], [28], [29], [30]. It can be directly performed on sputum samples of individuals with suspected TB, and can deliver results within 2 hours [30]. On the contrary, the LPA is used on the smear-positive pulmonary samples and on cultures from smear negative pulmonary TB cases with the ultimate goal of rapid diagnosis of MDR-TB [24].

Along these lines, present study attempted to evaluate the performances and applicability of the LPA assay and the GeneXpert MTB/RIF test for the rapid and effective detection of drug resistant and MDR *M. tuberculosis* isolates in Bangladesh. The results of both the molecular diagnostic methods were compared with that of the conventional phenotypic drug susceptibility test (DST) to demonstrate the sensitivity, specificity and accuracy of LPA and GeneXpert methods together with their capacity to shorten the time required for TB diagnosis compared to that of the conventional method.

## Material and Methods

### Study settings

The study was carried out at National Tuberculosis Reference Laboratory (NTRL) of National Institute of Diseases of Chest and Hospital (NIDCH), Bangladesh. The NTRL has the facilities for the diagnosis of TB in Dhaka city and also handles a substantial number of patients with complications referred from other hospitals and Thana Health Complexes within the country. The laboratory is supervised and has been certified by Supranational Reference Laboratory (SRL), Antwerp, Belgium.

### Study population

A total of 300 patients with increased suspicion of drug resistance recommended by their physicians were enrolled in this study from May 2012 to April 2013. All samples were smear positive and were collected from patients of higher-risk categories including failure, relapse, under treatment, return after default and delayed converter cases. The foremost cases included (i) category-I (n = 134), (ii) category-II (n = 149), (iii) category-IV (n = 5) who were admitted into NIDCH for a standardized re-treatment regimen and monitored by a DOTS plus program, and (iv) new patients (n = 12). All patients were tested for drug resistance within the mentioned time frame.

### Ethical issue

Permission was taken from the administrative authority of National Tuberculosis Reference Laboratory (NTRL) of National Institute of Disease the Chest and Hospital (NIDCH). Informed written consent from the patient or legal guardian was taken prior collecting samples. The NIDCH ethics committee specifically approved this study.

### Sample Collection, Smear preparation, staining and microscopic observation

Sputum samples were collected in clean, dry, wide-necked, leak-proof containers and smears were prepared from yellow purulent portion of the sputum using a sterile bamboo stick. The smear was spread evenly, air-dried for 15 minutes, and fixed by placing the slide over a hot plate at 85°C for 3 minutes [3], [6], [8]. For auramine O staining, the smear was covered with 0.1% auramine solution for 15 minutes, decolorized with 0.5% acid-alcohol for 3 minutes, and flooded by 0.3% methylene blue for 1 minute [6], [8]. After drying for 30 minutes, the smear was examined under light emitting diode (LED) fluorescence microscope at 455 nm (400× magnification) (Primo Star; Carl Zeiss LED, Germany).

### Conventional drug susceptibility test (DST)

Prior to DST, diagnosis by Lowenstein-Jensen (L-J) culture method was conducted. After decontamination and concentration of sputum samples following protocol previously described [3], [6], [8], 3–4 drops of the deposit were inoculated on two slopes of L-J media and incubated at 37°C. For the early recognition of rapidly growing *Mycobacterium* spp. and of contaminant microorganisms (if any), the media were examined within 3–5 days after inoculation. Culture was reported as positive one as soon as colonies of characteristic morphology constituting acid-fast bacilli were recognized [3], [5]. The isolates were identified by Auramin-O staining, growth rate, colony morphology, *p*-Nitrobenzoic Acid (PNB) susceptibility, catalase test, and nitrate reduction test [3], [5], [8].

For performing DST, a sterile, small thick walled screw-capped glass tube containing 5–7 sterile glass beads and tween 80 solution were taken, and one loopful colony, scraped from L-J culture media, was gently introduced over the beads and swirled. The tube was kept static for 15–30 minutes which allowed settling of the large aggregates of bacteria. Another tube containing 4.5 ml tween 80 solution was transferred (2 ml) to the homogenous upper part of the supernatant with similar dimension of the MacFarland standard for the visual comparison with the standard [3], [4], [8].

Serial dilution of the bacterial suspension was made up to 10^−5^. Bacterial suspensions were introduced in drug free medium to be used as growth control (GC). Tubes containing the drugs isoniazid (0.2 µg/ml) and rifampicin (40 µg/ml) were then inoculated with bacterial suspension from dilution 10^−3^ and dilution 10^−5^ [3], [5].

### Line probe assay (LPA)

#### DNA extraction

An aliquot of 500 µl of each of the decontaminated sputum samples was transferred into the 1.5 ml centrifuge tube. After the first centrifugation at 11,700 g for 15 minutes, the pellet was suspended into 100 µl of the yellow lysis buffer (A-LYS), heated at 100 °C for 10 min, and the resultant lysate was spun down. An equal volume (approximately 100 µl) of the neutralization buffer (A-NB) was added to the lysate and mixed well. The neutralized lysate was then centrifuged at 11,700 g for 5 minutes. Finally, around 100 µl of the supernatant (DNA) was harvested into an Eppendorf tube, from where 5 µl was used for amplification through the gene specific polymerase chain reaction (PCR) [24], [31], [32].

#### Master mix preparation

The master mix (amplification mix A, AM-A) consisted of 10× buffer (5 µl PCR buffer containing 15 mM MgCl_2_), oligonucleotides (2 µl mixture of ddATP, ddGTP, ddCTP and ddTTP supplemented with 25 mM MgCl_2_), DNA polymerase (0.2 µl *Taq* polymerase) and 2.8 µl dH_2_O. Amplification mix B (AM-B) consisted of MgCl_2_, the biotinylated primers (Table 1) and dye [33], [34]. For one PCR reaction, 10 µl of reagent-A was mixed with 35 µl of reagent-B and was mixed gently by inverting the tube for several times [31], [32].

**Table 1:** Primers for PCR amplification of specific genes in LPA method [33], [34].

Amplicon	Forward primer	Reverse primer
*rpoB* (81-base pair hyper-variable region)	5′-CGACCACTTCGGCAACCG-3′	3′-TCGATCGGGCACATCCGG-5′
*katG* (codon 315)	5′-TCGGCGGTCACACTTTCGGTAAGA-3′	3′-GCGACGCGTGATCCGCTCATAG-5′
*inhA* (promoter)	5′-CGAGCGTAACCCCAGTGCGAAAGT-3′	3′-CCCCGGTGAGGTTGGCGTTGAT-5′

#### Amplification and hybridization

An aliquot of 5 µl of each DNA template was added to the corresponding tube containing 45 µl of master mix and mixed gently. The PCR tubes were placed into the thermal cycler. The Genotype MTBDR assay was carried out according to the manufacturers’ instructions using the reagents provided in the kits (Hain Life Sciences, Germany). The process of PCR amplification, hybridization of the PCR products to the probe-containing strips, and detection and interpretation of the results were clearly described in the instruction [31], [35], [36].

### GeneXpert MTB/RIF

After collecting the sputum sample from a patient, sample reagent was mixed, and the suspension was shaken vigorously for 10–20 times. The resulting specimen was incubated at room temperature for 10 minutes. Again the specimen was shaken vigorously for 10–20 times and afterwards incubated at room temperature for 5 min. After removing the MTB/RIF cartridge (Version 3) from the Xpert machine (Cepheid, USA) the liquefied sample was aspirated by means of the sterile transfer pipette. Then the cartridge lid was opened and 2 ml of sample was transferred into the open port of the Xpert MTB/RIF cartridge. Finally the cartridge was placed on the Xpert machine and results were recorded within 2 hours [24], [27], [37].

## Results

### Frequency of drug resistant isolates

Among 300 samples, 292 were found to be culture positive and 3 experienced contamination. Two hundred and seventy seven (277) isolates were detected to be *Mycobacterium tuberculosis*, whereas 15 were found to be non-tubercular mycobacteria (NTM) as revealed from microscopic observation of the culture positive isolates (Table 2). Among the 277 isolates which were subjected to DST, 189 and 191 isolates exhibited resistance against rifampicin (RIF) and isoniazid (INH), respectively. Collectively 193 isolates were found to be drug resistant among which 187 were multidrug resistant (MDR) as they showed resistance against both RIF and INH (Table 2).

**Table 2:** Comparative analysis of frequency of MDR and mono drug resistant isolates estimated by the conventional drug susceptibility test (Solid culture DST) and LPA method.

		Conventional DST							Total
		RIF^R^ INH^R^	RIF^S^ INH^R^	RIF^R^ INH^S^	RIF^S^ INH^S^	*Not done	Contamination	NTM	
**LPA**	**RIF^R^ INH^R^**	186	0	1	0	0	2	0	189
	**RIF^S^ INH^R^**	0	4	0	0	0	0	3	7
	**RIF^R^ INH^S^**	1	0	1	0	0	0	0	2
	**RIF^S^ INH^S^**	0	0	0	84	0	1	0	85
	**Negative**	0	0	0	0	5	0	12	17
**Total**		187	4	2	84	5	3	15	**n = 300**

RIF = Rifampicin

INH = Isoniazid

RIF^R^ INH^R^ = MDR-TB.

RIF^S^ INH^R^ = INH mono resistant

RIF^R^ INH^S^ = RIF mono resistant

RIF^S^ INH^S^ = Both RIF & INH susceptible

NTM = Non Tubercle Mycobacterium

*Culture negative since no growth was observed on LJ media.

On the other hand, 198 drug resistant isolates were detected by LPA method among which 189 were found to be MDR as revealed from the detection of mutations in the genes responsible for the associated drug resistance (Table 2). One hundred and ninety one (191) and 196 isolates were found to be resistant against RIF and INH. Negative results were found for 17 isolates. Slightly extended resistance was found against RIF (n = 193) through the GeneXxpert assay compared to those from the conventional DST and LPA (Table 3 & 4). A total of 16 samples showed negative results, while 3 were found to be indeterminate.

**Table 3:** Comparative results of GeneXpert with conventional DST to determine the rifampicin (RIF) resistant/sensitive *M. Tuberculosis*.

		Conventional DST					Total
		Resistant	Sensitive	Not done	NTM	Contamination	
**GeneXpert**	**Resistant**	188	2	0	1	2	193
	**Sensitive**	1	85	0	2	0	88
	**Negative**	0	0	4	12	0	16
	**Indeterminate**	0	1	1	0	1	3
**Total**		189	88	5	15	3	**n = 300**

NTM = Non Tubercle *Mycobacterium.*

**Table 4:** Comparative results of LPA with GeneXpert for rifampicin resistance.

		LPA			Total
		Resistant	Sensitive	Negative	
**GeneXpert**	**Resistant**	190	3	0	193
	**Sensitive**	1	87	0	88
	**Negative**	0	0	16	16
	**Indeterminate**	0	2	1	3
**Total**		191	92	17	300

Patients under the category IV treatment (n = 5) were found to be MDR (Table 5). 47.8% and 48.5% MDR isolates were found by conventional DST and LPA methods, respectively in category I patients. Comparatively, the frequency of MDR was found to be higher in the category II patients as estimated to be 79.2% and 78.6% in LPA and conventional DST methods, respectively (Table 5). On the other hand, only 8.3% MDR cases were detected among the new TB cases by the above mentioned methods. Relatively higher frequency of resistance against RIF was also observed by the GeneXpert method compared to the other two methods for all the categories (Table 5).

**Table 5:** Comparison between conventional DST, LPA and GeneXpert methods in accordance with the category of the patients (n = 300).

Category			LPA					DST					GeneXpert		
			RIF	INH	MDR	Ng	RIF	INH	MDR	NTM	ND	Cont.	RIF	Ng	Ind.
**Cat-I (n = 134)**	**Fail (n = 66)**	**R**	44	46	43	0	44	45	43	0	0	1	44	0	1
		**S**	22	20			21	20					21		
	**RL (n = 16)**	**R**	8	9	8	1	8	8	8	1	0	0	9	1	0
		**S**	7	6			7	7					5		
	**UT (n = 35)**	**R**	8	8	8	4	7	8	7	4	0	0	8	3	1
		**S**	23	23			24	23					23		
	**RD (n = 5)**	**R**	3	3	3	0	3	3	3	0	0	0	3	0	0
		**S**	2	2			2	2					2		
	**DC (n = 12)**	**R**	3	3	3	2	3	2	3	3	0	0	3	2	0
		**S**	7	7			6	7					7		
**Cat-II (n = 149)**	**Fail (n = 92)**	**R**	76	77	76	0	75	75	75	2	0	1	76	0	0
		**S**	16	15			14	14					16		
	**RL (n = 23)**	**R**	18	18	18	1	18	18	18	1	0	0	18	1	1
		**S**	4	4			4	4					3		
	**UT (n = 26)**	**R**	18	18	18	3	18	18	18	3	1	0	18	3	0
		**S**	5	5			4	4					5		
	**RD (n = 2)**	**R**	2	2	2	0	2	2	2	0	0	0	2	0	0
		**S**	0	0			0	0					0		
	**DC (n = 6)**	**R**	4	4	4	0	4	4	4	0	0	0	4	0	0
		**S**	2	2			2	2					2		
**Cat-IV (n = 5)**		**R**	5	5	5	0	5	5	5	0	0	0	5	0	0
		**S**	0	0			0	0					0		
**New (n = 12)**		**R**	2	3	1	6	2	2	1	2	4	1	2	6	0
		**S**	4	3			4	4					4		

LPA =  Line Probe Assay; DST =  Drug Sensitivity Test; RIF =  Rifampicin; INH =  Isoniazid; R =  Resistant; S =  Sensitive; Ng. =  Negative; NTM =  Non-Tubercle Mycobacterium; ND =  Not done; Cont. =  Contamination; Ind. =  indeterminate; Cat-I =  Category-I; Cat-II =  Category-II; Cat-IV =  Category-IV; New =  New case; n =  number; Fail. =  Failure; RL =  Relapse; UT =  Under treatment; RD =  Return after Default; DC =  Delayed Converter.

### Efficacy of molecular techniques over conventional DST

With the aim of introducing molecular techniques for the detection of drug resistant tuberculosis in Bangladesh, the efficacy of LPA and Gene Xpert methods were evaluated. The LPA method was found to be 99.5%, 98.8% and 99.3% sensitive, specific and accurate, consecutively in detecting MDR-TB when compared to the conventional DST (Table 6). Besides, 99.5% sensitivity was observed for the detection of resistance against RIF and INH. GeneXpert method also showed 99.5% sensitivity in the detection of resistance against RIF when compared to those resulted from the conventional DST and LPA methods (Table 7). The GeneXpert method was 98.8% and 98.9% accurate when compared to conventional DST and LPA, respectively. On the other hand, the accuracy of LPA method for the detection of rifampicin (RIF) and isoniazid (INH) resistant isolates was 99.6% and 99.3%, respectively (Table 6). The positive and negative predictive values were also noticed to be higher in both the molecular diagnosis methods for the identification of individual drug resistance (both for RIF and INH) as well as for the detection of MDR cases (Tables 6 & 7).

**Table 6:** Performance assay of LPA method by comparing the results with that of the conventional DST (n = 277).

	Rifampicin resistance	Isoniazid resistance	Multi-drug resistance
**True resistant (n)**	188	190	186
**True sensitive (n)**	88	85	89
**Discordant (n)**	1	2	2
**Sensitivity (%)**	99.5	99.5	99.5
**Specificity (%)**	100	98.8	98.8
**Accuracy (%)**	99.6	99.3	99.3
**Positive predictive value (%)**	100	99.5	99.5
**Negative predictive value (%)**	98.9	98.8	98.8

Comparison was carried out among 277 isolates those were subjected to conventional solid culture DST.

**Table 7:** Performance assay of GeneXrept method when compared to LPA and Conventional- DST in detecting rifampicin (RIF) resistant isolates.

	1LPA	2Conventional DST
**Sensitivity (%)**	99.5%	99.5%
**Specificity (%)**	96.7%	97.7%
**Accuracy (%)**	98.6%	98.9%
**Positive predictive value (%)**	98.5%	98.9%
**Negative predictive value (%)**	98.9%	98.8%

1 Comparison was carried out among 281 isolates.

2 Comparison was carried out among 276 isolates.

## Discussion

Early detection of MDR-TB is crucial both for the patient management and infection control in TB positive cases [38]. Increased prevalence of drug resistant tuberculosis in Bangladesh and other developing countries is a growing threat to tuberculosis control since a few drugs has so far been found to be effective against TB [3], [4], [8], [38], [39], [40], [41], [42], [43]. According to the WHO report in 2010, the estimated MDR-TB among the notified retreatment cases (n = 7795) was 2200 (28%) [44]. Substantial increase in the global incidence of drug resistant TB cases raised the need for a more rapid and effective drug resistant TB detection method for the initiation of early and proper treatment of the patients and hence for the effective management of TB control program [45]. Therefore, present study endeavored to establish the molecular diagnostic methods, i.e., LPA and GeneXpert, to detect the mutation in specific genes responsible for the drug-resistance and also to accomplish the diagnosis procedure within a very short time. Commencing such a rapid and effective diagnostic method for the routine detection of drug resistant tuberculosis would no doubt be able to initiate proper treatment of the populations with the risk of TB propagation.

The findings of the present study indicated that the molecular methods were highly consistent with the conventional culture and DST method. Low frequency of discordant results and higher sensitivity in mono-drug and multi-drug resistance detection by the LPA and GeneXpert methods would be supportive of this fact. The methods could almost successfully detect the mutation in *rpoB* gene (responsible for RIF resistance) as 99.5% sensitivity was estimated when compared to that of the conventional DST. The specificity and accuracy were also found to be higher than those from the conventional diagnostic methods. Previous findings using LPA or Genotype MTBDR plus assay and also the GeneXpert assay for the detection of RIF resistance supported our data [18], [24], [45], [46], [47], [48].

Similar consistency was noticed for INH resistance as well as for MDR detection by LPA method. The sensitivity for INH detection was also found to be 99.5% which actually indicated the effective detection of mutations in *inh*A and *kat*G genes by LPA method. Detection of one of these genes individually could encounter significant variations in INH resistance detection as has been reported in the previous studies [21], [47], [49], [50]. Moreover, if only the mutation in *katG* or *inhA* were detected, a significant number of MDR-TB cases would have been missed out as happened in some other studies which detected the mutations only in the *kat*G gene [51], [52], [53]. Additionally, detection of the extended frequency of the resistant isolates along with the MDR ones as has been found in the current study is indeed evidently suggestive of the efficient performance of LPA and GeneXpert methods over the conventional DST.

Interestingly in our study, 2 of the MDR TB cases were found through the LPA method, which were also detected by GeneXpert as the RIF resistant ones, were surprisingly considered to be contamination in the conventional DST. A similar event has been noticed by a recent study by Barnard et al. (2008) [18]. On the other hand, 3 of the RIF resistant isolates found in LPA method were assumed to be non-tubercular mycobacteria (NTM) in the conventional DST method and one such case was also detected by the GeneXpert method. Such discrepancy might focus on the possibility of the onset of the false positive results by the molecular diagnostic methods although the frequency of such ambiguous cases was very negligible. However, the study findings were also found to be consistent according to the categories of the patients since higher frequency of drug resistance was observed among the category-IV and -II patients as had been anticipated.

Overall, the molecular methods are rapid, reliable and easy to interpret, although these techniques require more technical expertise. The LPA method takes not more than two days for the final interpretation of results starting from specimen selection, while GeneXpert can deliver results in less than two hours [18], [24], [45]. For both cases, the results of diagnosis thus have been found to be significantly shorter than the conventional DST method. Moreover, the molecular methods are cost effective as the cost of such assay is less than 50% of the conventional culture and DST methods [18]. Considering their accuracy or efficacy, rapidity and cost effectiveness, the molecular methods endorsed the aptitude to be implemented in the routine TB diagnosis laboratories all over the country for the effectual jurisdiction of the MDR-TB cases.
